# Supplementary material for: Measuring entanglement entropy and its topological signature for phononic systems
Source: Nat Commun. 2024 Feb 21;15:1601. doi: 10.1038/s41467-024-45887-8 (PMC10881961; doi:10.1038/s41467-024-45887-8)
Supplement: Supplementary file 1 — Supplementary Information [file 41467_2024_45887_MOESM1_ESM.pdf]

Supplementary Information, Lin *et al.*

Supplementary Note 1: Details of the design and fabrication of experimental samples ... 3

Supplementary Note 2: Details of the pump-probe response measurement for the phononic dispersions, wavefunctions, and reconstruction of nonlocal correlations 6

Supplementary Note 3: Reduced density matrix, entanglement entropy and entanglement spectrum in free-fermion systems 12

Supplementary Note 4: Widom conjecture of entanglement entropy in gapless free-fermion systems 15

Supplementary Note 5: Numerical simulation of entanglement entropy and entanglement spectrum 18

Supplementary Note 6: Numerical calculation in 1D and 2D concrete free-fermion systems 19

Supplementary Note 7: Probing band topology without relying on bulk-boundary correspondence and reliable indication of the topological transition even at small subsystem sizes 28

Supplementary Note 8: Details of the phononic edge dispersion measurement 30

Supplementary Note 9: Measurement of the Zak phase 30

Supplementary References 32

Supplementary Note 1: Details of the design and fabrication of experimental samples

In this work, phononic crystals are taken as a platform to study the entanglement entropy and entanglement spectrum. The elementary building blocks of the phononic crystals are the cylindrical acoustic cavities, of which the boundary is the hard wall for airborne sound waves. Such acoustic cavities support a number of acoustic eigenmodes with discrete eigenfrequencies. Here, we focus only on the lowest p_z-like eigenmodes and the phononic bands developed from such eigenmodes. With suitable geometric parameters designed in our experiments, the lowest p_z-like-orbital mode stays away from the other eigenmodes, as shown in Supplementary Fig. 1. The eigenfrequency of the p_z mode plays the role of the onsite potential when we map the phononic systems into tight-binding models. The additional coupling tubes connecting these cavities can shape the acoustic Su-Schrieffer-Heeger (SSH) and honeycomb lattices, which yield the corresponding phononic band structures. The radii of the tubes connecting the cylindrical acoustic cavities determine the acoustic coupling between these cavities. For the precision of the measurements we choose to work with the p_z-like phononic bands, instead of other phononic bands such as the s-like bands.

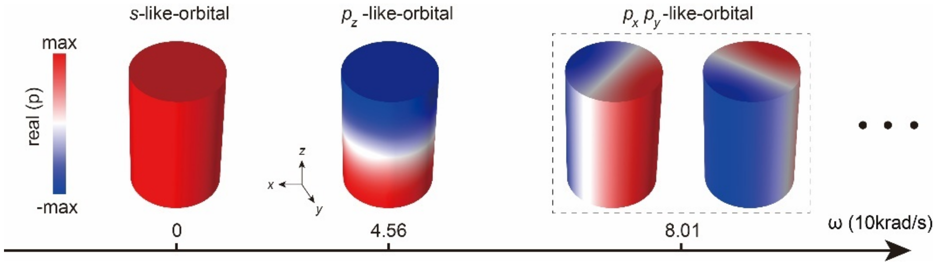

Supplementary Figure 1 | Lower-frequency eigenmodes of the single cylindrical resonator cavity with adopted geometric parameters in experimental SSH lattices. The p_z-like-orbital mode is chosen as the onsite frequency in this work to induce various phononic dispersions.

The unit cell structures for the experimental SSH and honeycomb lattices have been schematically illustrated in Figs. 2a and 3a in the main text, respectively. The major

geometric dimensions are also given in corresponding captions. Here, we summarize them again in Supplementary Tab. 1 for reference.

I

Variable	Geometric explanation	Dimension in [mm]
a	lattice constant	40
h	height of cylinder cavities	24
d	diameter of cylinder cavities	16
r_2	radii of the inter-unit-cell coupling tubes	4
r_1	radii of the intra-unit-cell coupling tubes	1.3/2/2.7/3.3/3.8/4/4.3/4.8/5.5

II

Variable	Geometric explanation	Dimension in [mm]
a	lattice constant	40
h_1	height of one cylinder cavity	24
h_2	height of the other cylinder cavity	24/28
d	diameter of all cylinder cavities	16
r	radii of all the coupling tubes	4

Supplementary Table 1 | Detailed geometry dimensions of air-structure unit cells for the experimental SSH (**I**) and honeycomb (**II**) lattices.

As shown in Supplementary Tab. 1, with r_2 fixed, r_1 in the SSH lattice takes several values that exemplify five topological ($r_1 < r_2$), one gapless ($r_1 = r_2$), and three trivial phases ($r_1 > r_2$). In the honeycomb lattice, the heights of two cylindrical cavities, h_1 and h_2, are set to different values (24 and 28mm) to result in a gapped phase, as the height difference breaks the inversion symmetry that protects the Dirac point. Note that, these geometric parameters refer to the air structure shaped by hard walls. In reality, the phononic crystals are manufactured by 3D-printing technology using photosensitive resin, which has an additional thickness of 2mm. The unit cells for the 3D-printed SSH and honeycomb lattices are illustrated in Supplementary Figs. 2a and 2b, respectively, where the thickness is labeled. The resin can be regarded as the hard-wall boundaries for airborne sound waves, as there is a huge acoustic impendence mismatch between resin and air background, at least in the frequency regime we are interested in, i.e., about 40 krad/s ~ 60 krad/s. For the sake of measurement, there gets a hole (diameter 7mm) on top of each cylindrical cavity [see Supplementary Figs. 2a and 2b], which

accommodates the insertion of a microphone for signal detection and a loudspeaker for excitation. All holes are stopped up by rubber plugs except two cavities inserted by the microphone and loudspeaker. The plugs are obtained from 3D-printing as well, but using rubber, which is also analogous to the hard wall for sound waves. The key geometric parameters of plugs are given in Supplementary Fig. 2c. In experiments, a phononic SSH lattice consists of 40 unit cells (80 cavities) along one direction. Given the maximum size that the 3D printer can support, the honeycomb lattice is printed into a rectangle shape made up of 1220 cavities which can be decomposed into 20 rows of zigzag lines. Each zigzag line comprises 61 cavities. To show more details, we give the schematic diagrams of the experimental SSH and honeycomb lattices in Supplementary Fig. 3.

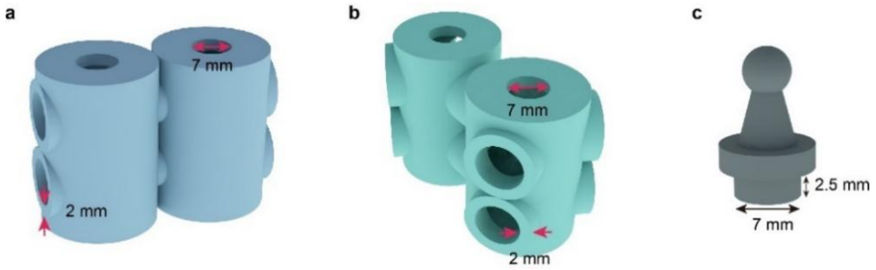

Supplementary Figure 2 | Illustration of the 3D-printed unit cells of the SSH (**a**) and honeycomb (**b**) lattices. The blue and green walls with a thickness of 2mm denote the photosensitive resin. The air encircled by resin walls is the air structure of unit cells that have been given in the main text. Each cylindrical cavity is designed to have a hole with a diameter of 7mm, for the sake of insertion of the microphone and loudspeaker. **c**. The 3D-printed rubber plug for stopping up the holes when cavities are not being detected.

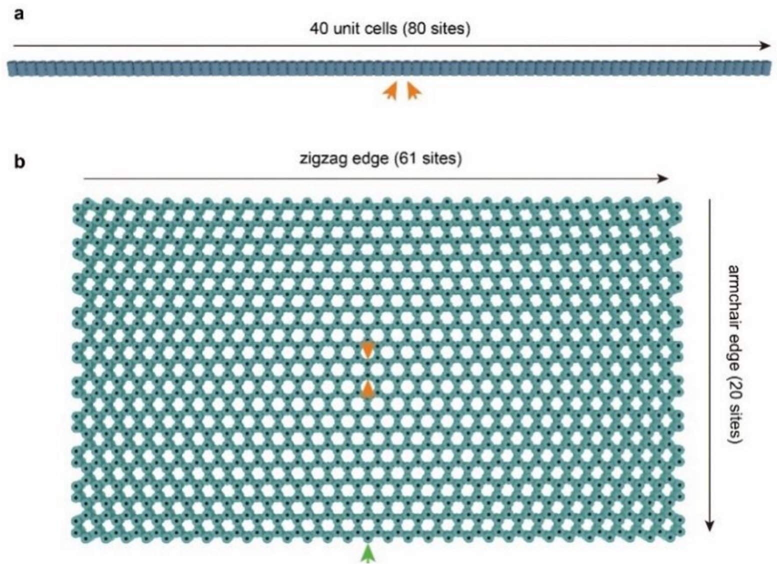

Supplementary Figure 3 | Illustration of the 3D-printed experimental SSH (**a**) and honeycomb (**b**) phononic crystals. The SSH lattice in (**a**) is composed of 40 unit cells with 80 cavities in total. The honeycomb lattice in (**b**) is printed into a rectangle shape with zigzag (61 cavities) and armchair (20 cavities) edge boundaries in the x- and y- directions, respectively. The orange (green) arrows point out the two positions of the small speaker for exciting the acoustic waves at different types of sites when performing the pump-probe measurements for the bulk (edge) phononic states.

Supplementary Note 2: Details of the pump-probe response measurement for the phononic dispersions, wavefunctions, and reconstruction of nonlocal correlations

The experimental results throughout this work are all derived from the pump-probe response measurement. In the measurement, the acoustic source is served as by a moving-coil-type loudspeaker with a diameter of 5mm. The loudspeaker is inserted through the hole into one cavity around the center of the phononic crystal, with a connection to a vector network analyzer (Keysight E5061B) that provides the signal stimulation. The excitation frequency is set as a regime to cover the measured phononic dispersions. For instance, the excitation frequency sweeps from 6.9 kHz to 8.6 kHz (4.3

krad/s ~ 5.4 krad/s) for the SSH lattice with $r_1 = 2$mm. The IF bandwidth in the network analyzer is set as 200 Hz, which is small enough for frequency resolution in our experiments. The power of the acoustic source has been amplified 10 times to improve the signal-to-noise ratio. The response within each cavity, including the amplitude and phase of the acoustic pressure field, is probed via a sub-wavelength microphone MC401 from BSWA TECH with a diameter of 6mm. The microphone is connected to the network analyzer as well, which records the detected response at the same time. In the process of the measurement, we fix the source situated at one cavity labeled by the coordinate site j and the sublattice site index α, and then detect the responses at all other cavities labeled by the coordinate i and the sublattice site index β. $\alpha, \beta = 1, 2$ for both the honeycomb lattice and the 1D lattice. By manually moving the microphone to traverse all cavities, the response tensor $\chi_{\alpha\beta}(i, j, \omega)$ is obtained. The pump-probe response can be measured and recorded for all possible sample configurations. In our experiments, for each SSH and honeycomb lattice, we measure twice the response functions with the loudspeaker fixed at one unit cell but two different sublattice sites. The loudspeaker situations for both the SSH and honeycomb lattices are highlighted by orange arrows in Supplementary Fig. 3. In these experiments, we record 1361 frequency points for each measurement.

We then apply the discrete Fourier transformation which acts on the unit cell coordinates, to acquire a 2×2 wavevector-space response tensor $\chi_{\alpha\beta}(\boldsymbol{k}, \omega)$ at each $\boldsymbol{k}$ and ω. The cavities closer to any edges have not been taken into consideration in the Fourier transformation to avoid spurious boundary effects. The response function $\chi_{\alpha\beta}(\boldsymbol{k}, \omega)$ is nearly proportional to the wavevector-space Green's function in the spectral representation,

$$\chi_{\alpha\beta}(\boldsymbol{k}, \omega) \propto \sum_{n\boldsymbol{k}} \frac{u_{n\boldsymbol{k}}^*(\alpha) u_{n\boldsymbol{k}}(\beta)}{\omega - (\omega_{n\boldsymbol{k}} + i\gamma_{n\boldsymbol{k}})}, \quad (1)$$

where $u_{n\boldsymbol{k}} = \begin{bmatrix} u_{n\boldsymbol{k}}(1) \\ u_{n\boldsymbol{k}}(2) \end{bmatrix}$ is the normalized periodic part of the unit-cell Bloch wavefunction corresponding to the eigenfrequency $\omega_{n\boldsymbol{k}}$. n and $\boldsymbol{k}$ denotes the band index and the Bloch wavevector, respectively. $\alpha, \beta = 1, 2$ is still the sublattice

index. $\gamma_{n\boldsymbol{k}}$ is a damping coefficient. It is apparent that the dominant contribution of the response function is from the resonance at eigenfrequency $\omega_{n\boldsymbol{k}}$. As a result, the phononic dispersions or the equifrequency contours can be directly reshaped from the intensity of response functions,

$$P(\boldsymbol{k},\omega) = \sum_{\alpha,\beta} \chi_{\alpha\beta}^{*}(\boldsymbol{k},\omega)\chi_{\alpha\beta}(\boldsymbol{k},\omega) \propto \sum_{n\boldsymbol{k}} \frac{1}{(\omega-\omega_{n\boldsymbol{k}})^2+\gamma_{n\boldsymbol{k}}^2}. \quad (2)$$

The normalization of $u_{n\boldsymbol{k}}$ is made use of by the above formula. Note that the intensity of the response function versus ω is analogous to a Lorenz curve depending on the damping coefficient $\gamma_{n\boldsymbol{k}}$ and the resonance frequency peak ω_{nk}. To exhibit the measured dispersion, the experimentally obtained $P(\boldsymbol{k},\omega)$ is given in Fig. 2c in the main text, where the SSH lattice with $r_1 = 2.7\ mm$ is taken as an example. The measured dispersions corresponding to other geometric parameters for SSH lattices are provided in Supplementary Fig. 4.

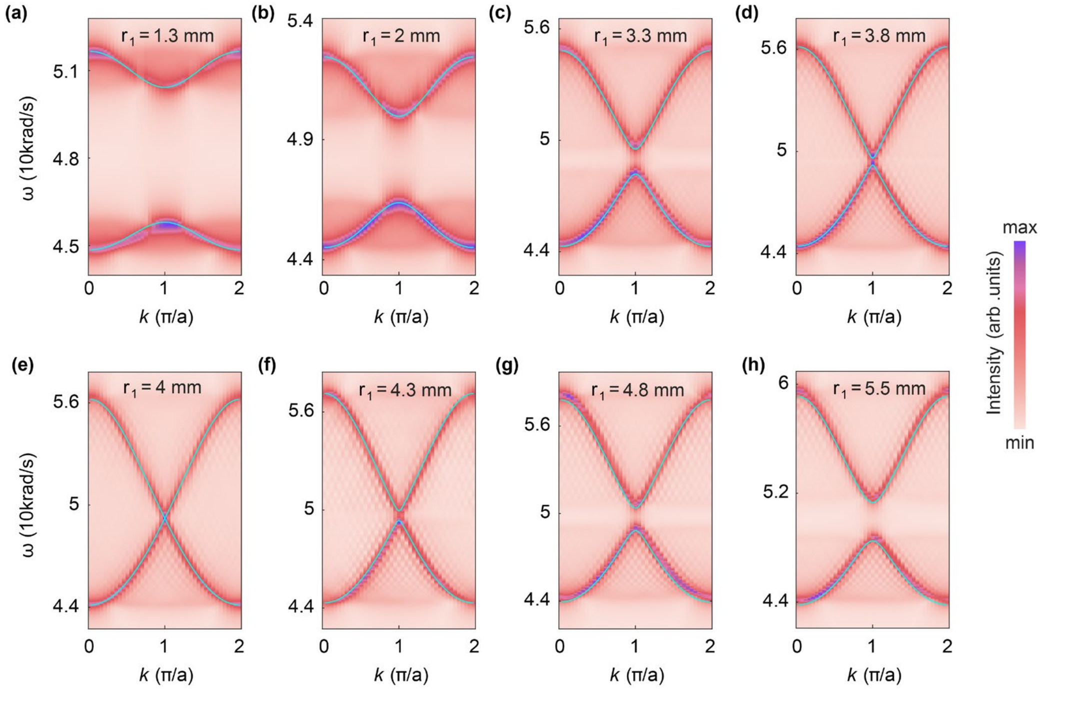

Supplementary Figure 4 | Experimentally measured dispersions for various 1D acoustic SSH chains. The radii of the intra-unit-cell coupling tube for these chains are (a) $r_1 = 1.3$mm, (b) $r_1 = 2$mm, (c) $r_1 = 3.3$mm, (d) $r_1 = 3.8$mm, (e) $r_1 = 4$mm, (f) $r_1 = 4.3$mm, (g) $r_1 = 4.8$mm, and (h) $r_1 = 5.5$mm, separately. Other parameters are $a = 40$mm, $h = 24$mm, $d = 16$mm, and $r_2 = 4$mm.

Some equifrequency contours of the honeycomb lattice in the gapless phase have also been presented in Fig. 3c in the main text. Here, we supply in Supplementary Fig. 5 the measured equifrequency contours of the gapped phase. All measured results regarding both the SSH and honeycomb lattices show a good agreement with calculated ones. We remark that the slight deviations between the experimental results and calculated curves are due to the actual fabrication precision, the finite-size effects of experimental samples, and the intrinsic dissipation of the acoustic waves. For instance, the finite-sample-size effects and the dissipation of the acoustic waves result in, respectively, the broadening of the phonon dispersion $\omega_{n\boldsymbol{k}}$ and wavevector $\boldsymbol{k}$. In experiments, we discretize the wavevector $\boldsymbol{k}$ into 40 inequivalent points in the first Brillouin zone for the 1D SSH lattice and 40×40 mesh points in the rhombic Brillouin zone for the 2D honeycomb lattice.

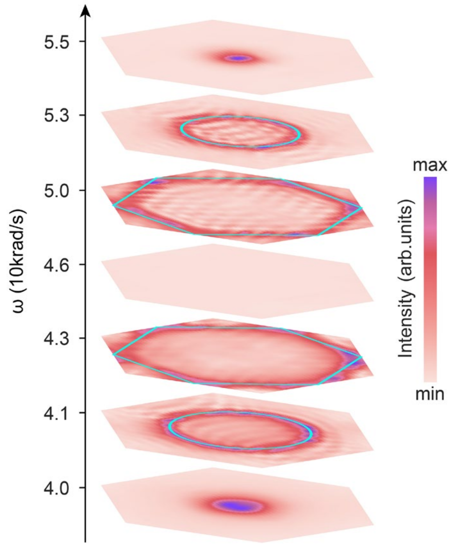

Supplementary Figure 5 | Experimentally measured equifrequency contours at certain frequencies of the honeycomb lattice in the gapless phase. All of them agree well with the equifrequency contours calculated from full-wave simulation (cyan curves).

According to Eq. (2), the response intensity $P(\boldsymbol{k}, \omega)$ has Lorentzian resonant shapes around each resonance peak. We thus fit the response intensity $P(\boldsymbol{k}, \omega)$ curve

with Lorentzian functions to extract the resonant frequency $\omega_{n\boldsymbol{k}}$ and the broadening of the resonance $\gamma_{n\boldsymbol{k}}$. We provide some examples in Supplementary Fig. 6 of the fitted curves where $\omega_{n\boldsymbol{k}}$ and $\gamma_{n\boldsymbol{k}}$ can be extracted which show the effectiveness of our spectral analysis.

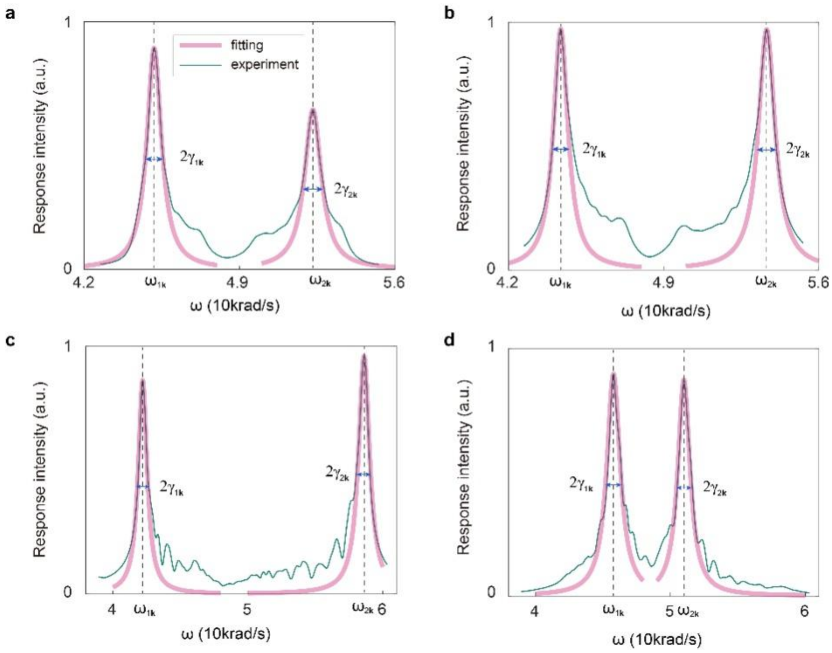

Supplementary Figure 6 | Fitted Lorentz curves (pink color) of the response intensity $P(\boldsymbol{k},\omega)$ (green curves) at (**a**) $k=\frac{\pi}{20a}$, (**b**) $k=\frac{9\pi}{40a}$, for the SSH lattice with $r_1=2.7\,mm$ and (**c**) $(k_1,k_2)=(0,\frac{K_2}{20})$, (**d**) $(k_1,k_2)=(\frac{19}{40}K_1,\frac{39}{40}K_2)$, for the honeycomb lattice in the gapless phase. K_1 and K_2 denote the length of two sides of the rhombus Brillouin zone. By fitting the two peaks,the corresponding eigenfrequency $\omega_{n\boldsymbol{k}}$ and the damping coefficient $\gamma_{n\boldsymbol{k}}$ can be deduced for both the first and second bands.

Furthermore, to obtain the phononic Bloch wavefunctions for the bulk states, we invent a spectral decomposition method for the analysis of the response tensor. A key observation based on Eq. (1) is that the measured response tensor $\chi_{\alpha\beta}(\boldsymbol{k},\omega_{n\boldsymbol{k}})$ at the eigenfrequency $\omega_{n\boldsymbol{k}}$ for a given band $n$and wavevector $\boldsymbol{k}$ is dominated by the term

$u_{n\boldsymbol{k}}^*(\alpha)u_{n\boldsymbol{k}}(\beta)$. Therefore, the 2×2 response tensor is nearly proportional to the covariance matrix,

$$\begin{bmatrix} u_{n\boldsymbol{k}}^*(1) \\ u_{n\boldsymbol{k}}^*(2) \end{bmatrix} [u_{n\boldsymbol{k}}(1), u_{n\boldsymbol{k}}(2)]. \tag{3}$$

To extract the Bloch eigenstate $u_{n\boldsymbol{k}}$, the response tensor $\chi(\boldsymbol{k}, \omega_{n\boldsymbol{k}})$ is then decomposed via singular value decomposition (SVD) into the following form,

$$\chi(\boldsymbol{k}, \omega_{n\boldsymbol{k}}) = U(\boldsymbol{k}, \omega_{n\boldsymbol{k}})\Sigma(\boldsymbol{k}, \omega_{n\boldsymbol{k}})V^\dagger(\boldsymbol{k}, \omega_{n\boldsymbol{k}}). \tag{4}$$

Here $U(\boldsymbol{k}, \omega_{n\boldsymbol{k}})$ and $V(\boldsymbol{k}, \omega_{n\boldsymbol{k}})$ are the left and right 2×2 eigenvector matrices, respectively. $\Sigma(\boldsymbol{k}, \omega_{n\boldsymbol{k}})$ is a 2×2 diagonal matrix, whose diagonal elements are non-negative singular values that are sorted from large to small. The first column of the left singular matrix $U(\boldsymbol{k}, \omega_{n\boldsymbol{k}})$ corresponding to the largest singular value is nothing but the measured normalized Bloch eigenstate $\begin{bmatrix} u_{n\boldsymbol{k}}(1) \\ u_{n\boldsymbol{k}}(2) \end{bmatrix}$ at eigenfrequency $\omega_{n\boldsymbol{k}}$.

Based on the measured Bloch eigenstate $u_{n\boldsymbol{k}}$, the frequency-dependent correlation function $C^A(i,j,\omega)$ in subsystem A can be obtained as follows[8,13],

$$C_{\alpha\beta}^A(i,j,\omega) = \sum_{n\boldsymbol{k}} \delta(\omega - \omega_{n\boldsymbol{k}})\, \psi_{n\boldsymbol{k}}^*(i,\alpha)\psi_{n\boldsymbol{k}}(j,\beta) \tag{5}$$

where $\psi_{n\boldsymbol{k}} = u_{n\boldsymbol{k}}e^{i\boldsymbol{k}\cdot\boldsymbol{r}}$ is the Bloch wavefunction spreading the whole sample, $\boldsymbol{r}$ denotes the unit-cell center coordinate. We remark that, with $u_{n\boldsymbol{k}}$ being normalized, the constructed Bloch wavefunction $\psi_{n\boldsymbol{k}}$ should be multiplied by a normalization coefficient $1/\sqrt{N}$, where N depends on the discretization of the first Brillouin zone. To be concrete, N is set as 40 $(40\times 40 = 1600)$ for the SSH (honeycomb) lattices. From the correlation function, we can obtain the correlation matrix and then determine the entanglement entropy and entanglement spectrum.

Supplementary Note 3: Reduced density matrix, entanglement entropy and entanglement spectrum in free-fermion systems

To theoretically measure the quantum entanglement in free-fermion systems, we need to compute entanglement entropy and entanglement spectrum. entanglement spectrum, which is defined as the eigenvalues of the entanglement Hamiltonian matrix, can be obtained by computing the eigenvalues of either correlation matrix or overlap matrix[1, 2]. Then, the entanglement entropy can be directly determined by the entanglement spectrum. In this Supplementary Note, we will give a detailed derivation to analytically connect the computation of the entanglement entropy and entanglement spectrum to correlation matrix.

For a free-fermion system, its second-quantized Hamiltonian can be expressed in the following generic quadratic form:

$$H = \sum_{i,j} \hat{c}_i^\dagger \mathcal{H}_{i,j} \hat{c}_j, \tag{6}$$

where $i, j\ldots$ label the lattice sites as well as all other indices (e.g., spin, orbitals) at each site. $\hat{c}_i^\dagger(\hat{c}_j)$ is the fermionic creation (annihilation) operator and satisfies the anti-commutation relationship $\{\hat{c}_i, \hat{c}_j^\dagger\} = \delta_{i,j}$ where δ is a usual Kronecker symbol. The single-particle Hamiltonian matrix $\mathcal{H}$ can be diagonalized by introducing a set of ortho-normal eigenstates $\{|\alpha>\}$ that satisfy $<\alpha|\beta>= \delta_{\alpha,\beta}$, which leads to $\mathcal{H} = \sum_\alpha E_\alpha |\alpha><\alpha|$. As a result, H is transformed to the following diagonalized form:

$$H = \sum_\alpha E_\alpha \sum_i (\psi_\alpha^*(i) c_i)^\dagger \sum_j \left(\psi_\alpha^*(j) c_j\right) = \sum_\alpha E_\alpha \psi_\alpha^\dagger \psi_\alpha, \tag{7}$$

where $\hat{\psi}_\alpha^\dagger(\hat{\psi}_\alpha)$ is a fermionic creation (annihilation) operator such that $|\alpha>= \hat{\psi}_\alpha^\dagger|0>$, $\hat{\psi}_\alpha|0>= 0$, and $\{\hat{\psi}_\alpha, \hat{\psi}_\beta^\dagger\} = \delta_{\alpha,\beta}$. Here, $|0>$ denotes the vaccum state that is empty of fermions. On the other hand, the creation operators $\hat{c}_i^\dagger$ and $\hat{\psi}_\alpha^\dagger$ can be transformed into each other via

$$\hat{\psi}_\alpha^\dagger = \sum_i < i|\,\alpha > \hat{c}_i^\dagger = \sum_i \psi_\alpha(i)\, \hat{c}_i^\dagger,$$

$$\hat{c}_i^\dagger = \sum_\alpha < \alpha|\, i > \hat{\psi}_\alpha^\dagger = \sum_\alpha \psi_\alpha^*(i)\, \hat{\psi}_\alpha^\dagger. \quad (8)$$

Here $\psi_\alpha(i)$ is a wavefunction defined as $\psi_\alpha(i) =< i\,|\alpha >$. Then, the many-body ground state $|GS>$ of the original free-fermion system with N fermions can be directly constructed by acting $\hat{\psi}_\alpha^\dagger$ on the vacuum state: $|GS>= \prod_{\alpha\in occ.} \hat{\psi}_\alpha^\dagger|0>$, where "$\alpha \in$ occ." means that the N lowest energy levels are occupied. By means of the many-body ground state, the density matrix can be readily expressed as

$$\rho = |GS><GS|, \quad (9)$$

when we partition the total system into two subsystems (i.e., A and B) as shown in Fig. 1a in the main text. By tracing out the degrees of freedom in the subsystem B, we arrive at the reduced density matrix (RDM) $\rho_A = Tr_{i\in B}(\rho)$, which has the following Gaussian (quadratic) form for a free-fermion system[3]:

$$\rho_A = \frac{1}{Z} e^{-H^E}, H^E = \sum_{i,j\in A} \hat{c}_i^\dagger h_{i,j}^E \hat{c}_j. \quad (10)$$

Here, $Z = Tr(e^{-H^E})$ is a normalization constant and h^E is the entanglement Hamiltonian matrix. Let $\phi_n(i)$ be the eigenfunction of the entanglement Hamiltonian matrix h^E with eigenvalue ξ_n. Then, the fermionic operators $\hat{c}_i$ can be transformed to a new set of fermionic operators $\hat{a}_n$ via:

$$\hat{c}_i = \sum_n \phi_n(i)\, \hat{a}_n\, . \quad (11)$$

By using this transformation, ρ_A is thus sent to its diagonalized form:

$$\rho_A = \frac{1}{Z} e^{-\sum_{n,n'} \sum_i \left(\phi_n^*(i)\hat{a}_n^\dagger\right) h_{i,j}^E \sum_j (\phi_{n'}(j)\hat{a}_{n'})} = \frac{1}{Z} e^{-\sum_n \xi_n \hat{a}_n^\dagger \hat{a}_n}, \quad (12)$$

where the set of real numbers $\{\xi_n\}$ forms the single-particle entanglement spectrum, $\sum_{i,j} \phi_n(i)\phi_{n'}^*(j) = \delta_{n,n'}$, and $\sum_n \phi_n^*(i)\phi_n(j) = \delta_{i,j}$. In other words, h^E is transformed to

$$h_{i,j}^E = \sum_n \phi_n(i)\phi_n^*(j)\, \xi_n. \quad (13)$$

Next, we discuss the relation between correlation matrix and entanglement Hamiltonian matrix. For a free-fermion system, the definition of real-space (equal-time) correlation matrix is given by:

$$C_{i,j} =< GS|\hat{c}_i^\dagger \hat{c}_j|GS >. \tag{14}$$

If we further restrict i, j in the subsystem A, by applying Eq. (8), we express the correlation matrix (denoted as $C_{i,j}^A$) of the subsystem A in terms of ortho-normal eigenstates $\{|\alpha >\}$:

$$C_{i,j}^A =< GS|\hat{c}_i^\dagger \hat{c}_j|GS >= \sum_{\alpha \in occ.} C_\alpha^{single}(i,j), \tag{15}$$

where $C_\alpha^{single}(i,j)$ is the single-particle correlation matrix defined as $C_\alpha^{single}(i,j) =< \alpha|\hat{c}_i^\dagger \hat{c}_j|\alpha >= \psi_\alpha^*(i)\psi_\alpha(j)$. Alternatively, by means of the density matrix ρ, the correlation function defined in Eq. (14) can be rewritten as:

$$C_{i,j} = Tr_{A,B}(\rho \hat{c}_i^\dagger \hat{c}_j) = Tr_A[Tr_B(\rho \hat{c}_i^\dagger \hat{c}_j)]. \tag{16}$$

Here, $Tr_{A(B)}$ denotes the trace operation of the subsystem A (B) . Therefore, when $i, j \in A$, the correlation matrix $C_{i,j}^A$ can be reexpressed as

$$C_{i,j}^A = Tr_A(\rho_A \hat{c}_i^\dagger \hat{c}_j). \tag{17}$$

Here, the reduced density matrix $\rho_A = Tr_B(\rho)$ has been introduced in Eq. (10). Combined with Eq. (12) and $Tr_A(\rho_A) = Tr_A(Tr_B(\rho)) = Tr_{A,B}(|GS >< GS|) = 1$, the correlation matrix $C_{i,j}^A$ restricted in the subsystem A reduces to:

$$C_{i,j}^A = Tr_A\left(\frac{1}{Z} e^{-\sum_{n'} \xi_{n'} \hat{a}_{n'}^\dagger \hat{a}_{n'}} \sum_n \phi_n^*(i)\phi_n(j)\hat{a}_n^\dagger \hat{a}_n\right)$$

$$= \sum_n \phi_n^*(i)\phi_n(j) Tr_A\left(\frac{1}{Z} e^{-\sum_n \xi_n \hat{a}_n^\dagger \hat{a}_n} \hat{a}_n^\dagger \hat{a}_n\right)$$

$$= \sum_n \phi_n^*(i)\phi_n(j) \frac{1}{e^{\xi_n+1}}. \tag{18}$$

At this point, it is straightforward to verify the following eigenequation: $\sum_j C_{i,j}^A \phi_n(j)$=$\frac{1}{e^{\xi_n+1}} \phi_n(i)$ by taking advantage of the ortho-normal property of $\phi_n(i)$. Therefore, $\{\phi_n(i)\}$ exactly forms the set of eigenfunctions of the correlation matrix $C_{i,j}^A$, and the associated eigenvalues (denoted as ε_n) are fully determined by the single-particle entanglement spectrum (denoted as ξ_n) via the following identity:

$$\varepsilon_n = \frac{1}{e^{\xi_n + 1}}, \tag{19}$$

In other words, by comparing Eq. (13) and (18), we find that the entanglement Hamiltonian matrix h^E and correlation matrix C^A can be simultaneously diagonalized.

In practice, due to the exact one-to-one correspondence between ε_n and ξ_n, ε_n, which is rigorously bounded inside a finite range $[0,1]$, is often adopted to represent the entanglement spectrum in the literature, although ξ_n is originally defined as the entanglement spectrum. Symbolically, the above relation between the two matrices, i.e., h^E and C^A, can be encoded in the following compact form

$$h^E = \log[(C^A)^{-1} - \mathbb{I}], \tag{20}$$

where $\mathbb{I}$ is the identity matrix.

In conclusion, the entanglement spectrum of a free-fermion system can be fully determined by diagonalizing the correlation matrix restricted in the subsystem A. Once we obtain $\{\varepsilon_n\}$, the entanglement entropy of the free-fermion system is reached:

$$S = -Tr\rho_A \log \rho_A = -\sum_n [\varepsilon_n \log \varepsilon_n + (1-\varepsilon_n)\log(1-\varepsilon_n)]. \tag{21}$$

From this expression, it is apparent that the maximal entanglement entropy contribution comes from all eigenmodes with $\varepsilon_n \sim 0.5$, which is exactly the zero modes of the entanglement Hamiltonian ξ_n according to Eq. (19).

Supplementary Note 4: Widom conjecture of entanglement entropy in gapless free-fermion systems

In this Supplementary Note, we start with the Schrödinger equation to analyze the correlation matrix and the Widom conjecture of entanglement entropy in gapless free fermion systems. To be specific, the Schrödinger equation in a lattice system is written as

$$i\partial_t \psi(i,t) = \mathcal{H}_{i,j}\psi(j,t), \tag{22}$$

where $\mathcal{H}$ is the Hamiltonian matrix defined in Eq. (6). Assuming the initial state $\psi(i,0) = \varphi(i)$, the time evolution of $\varphi(i)$ is represented as

$$\psi(i,t) = \sum_\alpha c_\alpha \psi_\alpha(i) e^{-iE_\alpha t}, \tag{23}$$

where the coefficient $c_\alpha = \sum_j \psi_\alpha^*(j)\varphi(i)$. Here $\psi_\alpha(i) =< i|\alpha >$ is the eigenfunction of $\mathcal{H}$ with eigenvalue E_α, which is introduced in Supplementary Note 3. It is also known that the retarded Green's function is defined as

$$iG(i,j,t) \equiv< i\left|e^{-i\mathcal{H}t}\right|j >. \tag{24}$$

Then, the Green's function also satisfies the Schrödinger equation as

$$i\partial_t G(i,j,t) = \sum_j \mathcal{H}_{i,j} G(i,j,t) \tag{25}$$

with the initial condition $G(i,j,0) = -i\delta_{i,j}$. Inserting the complete basis $\mathbb{I} = \sum_\alpha |\alpha >< \alpha|$ into the above equation, the Green's function is formally solved:

$$iG(i,j,t) = \sum_\alpha \psi_\alpha(i)\psi_\alpha^*(j) e^{-iE_\alpha t}. \tag{26}$$

Therefore, the wave function $\psi(i,t)$ on the i site becomes $\psi(i,t) = -\sum_j iG(i,j,t)\varphi(i)$. In the frequency space, the retarded Green's function becomes

$$G(i,j,\omega) = \int_{-\infty}^{\infty} dt G(i,j,t) e^{i\omega t - 0^+ t} = \sum_\alpha \frac{\phi_\alpha(i)\phi_\alpha^*(j)}{\omega - E_\alpha + i0^+}, \tag{27}$$

where $G(i,j,\omega)$ can be measured in the phononic platform. It is worth mentioning that the equal-time correlation function $C_{i,j}^A$ is distinct from the retarded Green's function and thus cannot be directly determined from the measured pump-probe response.

The correlation matrix C^A of a free-fermion system in subsystem A possesses another interesting property that is vital to the Widom conjecture to be introduced shortly. By introducing two projection operators $\hat{R} = \sum_{i\in A} |i >< i|$ and $\hat{P} = \sum_\alpha \theta(-\varepsilon_\alpha) |\alpha >< \alpha|$ that satisfy $\hat{R} = \hat{R}\hat{R}$ and $\hat{P} = \hat{P}\hat{P}$, the correlation matrix C^A can be alternatively expressed as[4, 5]

$$C^A = \hat{R}\hat{P}\hat{R}. \tag{28}$$

Here $|\alpha >$ and ε_α are respectively the ortho-normal eigenstates and eigenvalues of the Hamiltonian matrix $\mathcal{H}$ already introduced in Supplementary Note 3, and θ denotes the standard step function. Based on the expression of the entanglement

entropy in Eq. (21), we can apply two projection operators to rewrite the entanglement entropy in the following useful form:

$$S_A = -Tr\rho_A \log \rho_A = -\sum_n [\varepsilon_n \log \varepsilon_n + (1-\varepsilon_n)\log(1-\varepsilon_n)]$$

$$= \sum_n f(\xi_n) = Tr\left[f\left(\hat{R}\hat{P}\hat{R}\right)\right], \quad (29)$$

where the function f is defined as $f(t) = -t\log t - (1-t)\log(1-t)$. Eq. (29) indicates that the sum of function f acting on the spectrum of the Hermitian operator $\hat{R}\hat{P}\hat{R}$ equals to the trace of the function f acting on the Hermitian operator $\hat{R}\hat{P}\hat{R}$ [6]. When $L \to \infty$, the asymptotic behavior of $Tr[f(\hat{R}\hat{P}\hat{R})]$ is elegantly determined by the Widom conjecture[7]. By using the conjecture, the scaling of entanglement entropy is analytically represented as

$$S_A = Tr\left[f\left(\hat{R}\hat{P}\hat{R}\right)\right] = \frac{L^{d-1}}{(2\pi)^{d-1}}\frac{\log L}{12}\int_{\partial\Omega}\int_{\partial\Gamma}|\mathbf{n}_{\mathrm{x}}\cdot\mathbf{n}_{\mathrm{k}}|dA_x dA_k, \quad (30)$$

where $\partial\Gamma$ and $\partial\Omega$ are respectively the boundaries of the Fermi sea in the momentum space and the subsystem A in real space, $\mathbf{n}_{\mathrm{x}}$ and $\mathbf{n}_{\mathrm{k}}$ are unit normal vectors for these boundaries, as shown in Fig. 1a in the main text.

Next, we apply the Widom conjecture to derive the scaling behaviour of entanglement entropy in Eq. (29). For a general class of function f and $L \to \infty$, the asymptotic formula[6, 8] of the Widom conjecture is written as

$$Tr\left[f\left(\hat{R}\hat{P}\hat{R}\right)\right] = \left(\frac{L}{2\pi}\right)^d f(1)\int_\Omega\int_\Gamma d\mathbf{x}d\mathbf{k} +$$

$$\left(\frac{L}{2\pi}\right)^{d-1}\frac{\log 2\log L}{4\pi^2}U(f)\int_{\partial\Omega}\int_{\partial\Gamma}|\mathbf{n}_{\mathbf{x}}\cdot\mathbf{n}_{\mathbf{k}}|dA_x dA_k + o(L^{d-1}\log L), \quad (31)$$

where $\mathbf{n}_{\mathbf{x}}$ and $\mathbf{n}_{\mathbf{k}}$ are respectively unit normal vectors to $\partial\Omega$ and $\partial\Gamma$, and

$$U(f) = \int_0^1 \frac{f(t)-tf(t)}{t(1-t)}dt. \quad (32)$$

Γ and Ω are respectively the bulk of the Fermi sea and the bulk of the subsystem A. By using Eq. (30), we immediately arrive at the scaling behaviour of the entanglement entropy in a gapless free-fermion system with $(d-1)$-dimensional Fermi surface. It should be noted that, the Hermiticity of $\hat{R}\hat{P}\hat{R}$ is crucial in establishing Eq. (31).

In addition to the entanglement entropy and entanglement spectrum, mutual information[9] , is also an important entanglement quantity in both quantum many-body systems and quantum information. In terms of entanglement entropy, mutual information is defined as follows:

$$I(A\!:\!B) := S(A) + S(B) - S(AB). \quad (33)$$

Here A and B are two individual subsystems of the total system, and the symbol "AB" denotes the union of A and B. It should be noted that here AB does not cover the whole system. In practice, mutual information is commonly adopted to quantify mixed states. When ρ_{AB} is a density matrix of a pure state, i.e., $S(AB) = 0$ and $S(A) = S(B)$, the mutual information and entanglement entropy are related to each other via $I(A\!:\!B) = 2S(A)$.

Supplementary Note 5: Numerical simulation of entanglement entropy and entanglement spectrum

The way to derive the simulated entanglement entropy and entanglement spectrum is as follows: We first obtain the phonon eigenfrequency $\omega_{n\boldsymbol{k}}$ and the corresponding wavefunction $\psi_{n\boldsymbol{k}}$ by performing finite-element simulations based on the acoustic wave equation in the phononic crystal which in this work is done with the help of the software COMSOL Multiphysics. To compare with the experimental data, the Bloch wavevectors are discretized in the same way as in the experiments. At each n and $\boldsymbol{k}$, we extract the acoustic pressure fields at only two points in a unit cell to act as the unit-cell Bloch wavefunctions $u_{n\boldsymbol{k}}$. Because the correlation matrix does not include the information of dissipation directly, in the acoustic simulations we also do not include the dissipation effects.

With the $\omega_{n\boldsymbol{k}}$ and $\psi_{n\boldsymbol{k}}$ from the finite-element acoustic simulations, we can construct the correlation function according to Eq. (4) and then calculating the correlation matrix according to Eq. (1) in the main text. From the correlation matrix,

the entanglement entropy and entanglement spectrum can be obtained according to Eq. (2) in the main text. The results presented in the main text show a good agreement between simulations and experiments.

Supplementary Note 6: Numerical calculation in 1D and 2D concrete free-fermion systems

In this Supplementary Note, we numerically study the scaling behavior of entanglement entropy in 2D graphene and 1D SSH tight-binding model.

We first consider the tight-binding model of graphene, which is defined on a honeycomb lattice with two distinct atoms a and b per unit cell as shown in Supplementary Fig. 7a. The corresponding Hamiltonian matrix in momentum space reads:

$$H_0(\mathbf{k}) = \begin{pmatrix} 0 & h(\mathbf{k}) \\ h(\mathbf{k}) & 0 \end{pmatrix} - \mu\sigma_0, \tag{34}$$

where $\mathbf{k} = (k_x, k_y)$, μ denotes the chemical potential and

$$h(\mathbf{k}) = t\sum_m e^{i\mathbf{k}\cdot\delta_m}. \tag{35}$$

Here the nearest neighbors on the lattice are connected by three vectors denoted as δ_m (m =1,2,3) and they share the same hopping energy t, as shown in Supplementary Fig. 7a. By diagonalizing the Hamiltonian matrix, the energy dispersion can be determined:

$$E(\mathbf{k}) = \pm|h(\mathbf{k})| - \mu. \tag{36}$$

According to Eq. (36), when we adjust the chemical potential μ, the shape of Fermi surface gradually changes. When $\mu = 0$, the Fermi surface is shrunk to two distinct Dirac points respectively located in $\mathbf{K} = (\frac{2\pi}{3}, \frac{2\pi}{3\sqrt{3}})$ and $\mathbf{K}' = (\frac{2\pi}{3}, \frac{-2\pi}{3\sqrt{3}})$, as shown in Supplementary Figs. 7c and 7d.

Next, we numerically study the entanglement entropy in graphene. We note that, when $L \to \infty$, the entanglement entropy in a d-dimensional free-fermion system with $(d-1)$-dimensional Fermi surface scales as[6,8,10,11]

$$S_A = cL^{d-1}\log L, \quad (37)$$

where the constant c can be analytically obtained from Eq. (31). We partition the graphene system into two parts A and B as demonstrated in Supplementary Fig. 7b, where the periodic boundary condition is considered. To calculate the entanglement entropy, it is sufficient to derive the following real-space equal-time correlation matrix in the subsystem A:

$$C^A(i,j) = \begin{bmatrix} < GS|\hat{c}_{a,i}^{\dagger}\hat{c}_{a,j}|GS > & < GS|\hat{c}_{b,i}^{\dagger}\hat{c}_{a,j}|GS > \\ < GS|\hat{c}_{a,i}^{\dagger}\hat{c}_{b,j}|GS > & < GS|\hat{c}_{b,i}^{\dagger}\hat{c}_{b,j}|GS > \end{bmatrix}, \quad (38)$$

where $\hat{c}_{a(b),i}$ denotes the annihilation operator of spinless fermions at the sublattice a (b) in the ith unit cell. Each entry of $C^A(i,j)$ can be determined by single-particle correlation matrix in Eq. (15) by adding extra sublattice indices. For example,

$$< GS|\hat{c}_{a,i}^{\dagger}\hat{c}_{a,j}|GS > = \sum_{\alpha\in occ.} C_{a,a}^{\alpha}(i,j). \quad (39)$$

Once the correlation matrix C^A is obtained, we can use its eigenvalues to calculate entanglement entropy according to Eq. (21).

As shown in Supplementary Figs. 7c and 7d, if the chemical potential $\mu = 0$, the Fermi surface of graphene is shrunk to two Dirac points. When $\mu \neq 0$, a 1-dimensional Fermi surface appears. As shown in Supplementary Fig. 8b, when 1-dimensional Fermi surface exists, the entanglement entropy scales as $S_A \sim cL \ln L$. As shown in Supplementary Fig. 8b, when the Fermi surface is shrunk to two Dirac points, S_A/L saturates to a constant in the scaling limit. In addition, we also explore the behavior of the constant c in Eq. (37) with different chemical potentials. We find the value of c monotonically depends on the size of Fermi surface. Meanwhile, we consider four shapes, including parallelogram, rectangle, triangle and rhmobus, to study the effect of different partitions for the EE. As shown in Fig. 8c, we find that the behaviour of the constant c in the scaling of EE with different chemical potential is not affected by the shape of subsystem A, thus satisfying the Widom conjecture. In Supplementary Fig. 8b, the numerical results with nonzero chemical potentials and thereby 1-dimensional FS provide a positive verification of the Widom conjecture in Eq. (30).

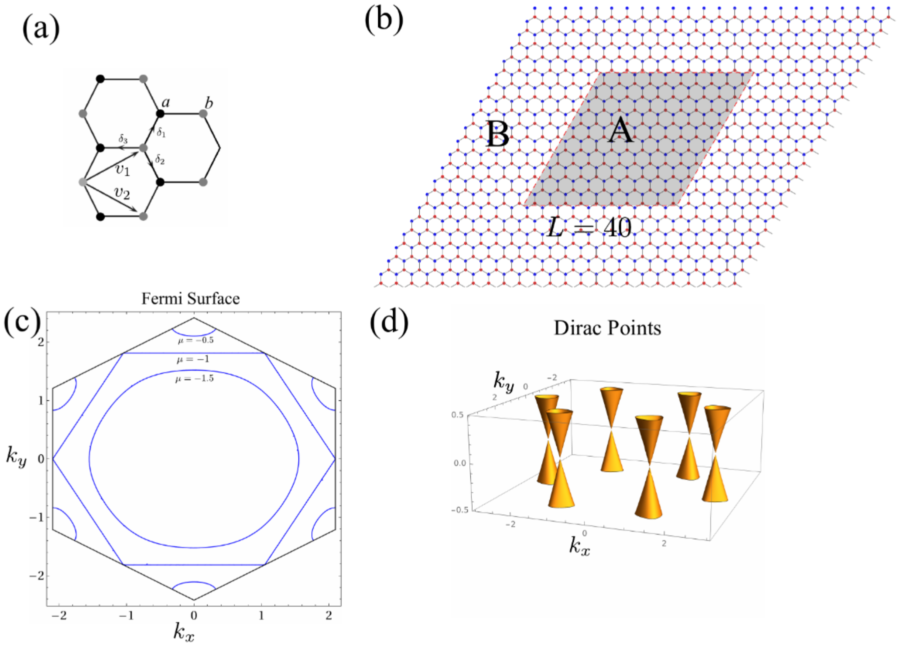

Supplementary Figure 7 | a, The graphene lattice. **b** is the partition of graphene**. c**, Fermi surface of graphene with different chemical potentials μ. **d**, The energy dispersion near Dirac points of graphene with $\mu = 0$.

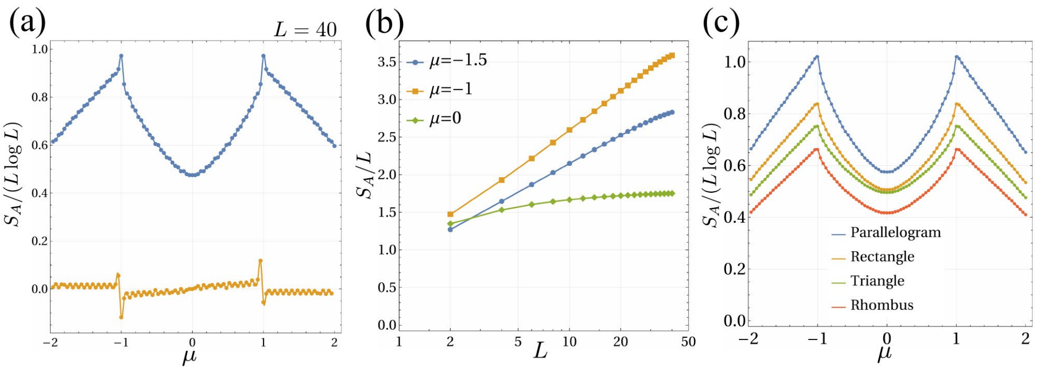

Supplementary Figure 8 | a, The blue line represents the behaviors of the constant c in Eq. (37) with different chemical potential μ and the fixed size of the subsystem, and the orange line represents the derivative of the constant c in Eq. (37). **b**, The scaling behavior of entanglement entropy in graphene where the horizontal axis is scaled to be logarithm of L. **c**, The illustration of the constant c in Eq. (37) with four kinds of partitions for different chemical potential μ.

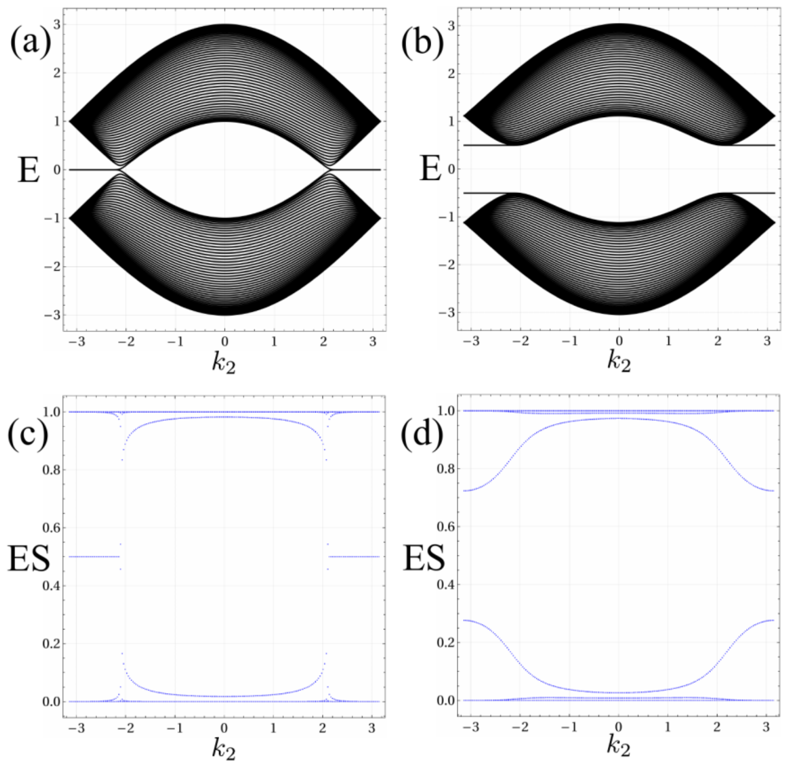

Supplementary Figure 9 | (**a**) and (**b**) are the energy spectrum of the graphene system with $m = 0$ and $m = 0.5$, respectively. (**c**) and (**d**) are the entanglement spectrum of the graphene system with $m = 0$ and $m = 0.5$, respectively.

After the entanglement entropy of graphene is numerically examined, we move on to the correspondence between the entanglement spectrum and the energy spectrum which has been previously theoretically analyzed in both gapped phases and gapless phases, e.g., topological insulators[12], and higher order Weyl semimetals[13]. According to the energy dispersion in Eq. (36), the energy spectrum is gapless. To show how a finite energy gap affects the entanglement spectrum, we may add a term $m\sigma_x$ on each site, where σ_x is a Pauli matrix and m is a positive constant. For the graphene system, its energy spectrum has zero modes with zigzag boundary condition along k_1 direction, as shown in Supplementary Fig. 9a. Furthermore, to demonstrate the entanglement spectrum of the graphene system, we use the spectrum of the correlation matrix in the subsystem A to represent

entanglement spectrum, as explained in Supplementary Note 3. In Supplementary Fig. 9c, we find the existence of $\frac{1}{2}$ modes in the entanglement spectrum which corresponds to the zero modes of the energy spectrum. When $m = 0.5$, the energy spectrum is gapped as shown in Supplementary Fig. 9b. Then, the entanglement spectrum does not have $\frac{1}{2}$ modes and is gapped. Therefore, the entanglement spectrum provides a clue for the experimental test of zero modes of energy spectrum.

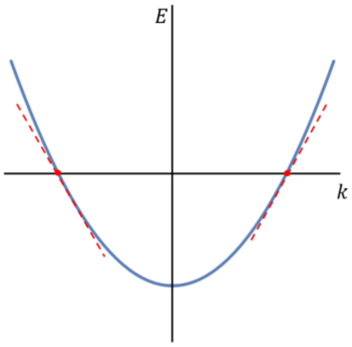

Supplementary Figure 10 | The energy dispersion of a one-band free-fermion system, this system has two gapless modes described by conformal field theory.

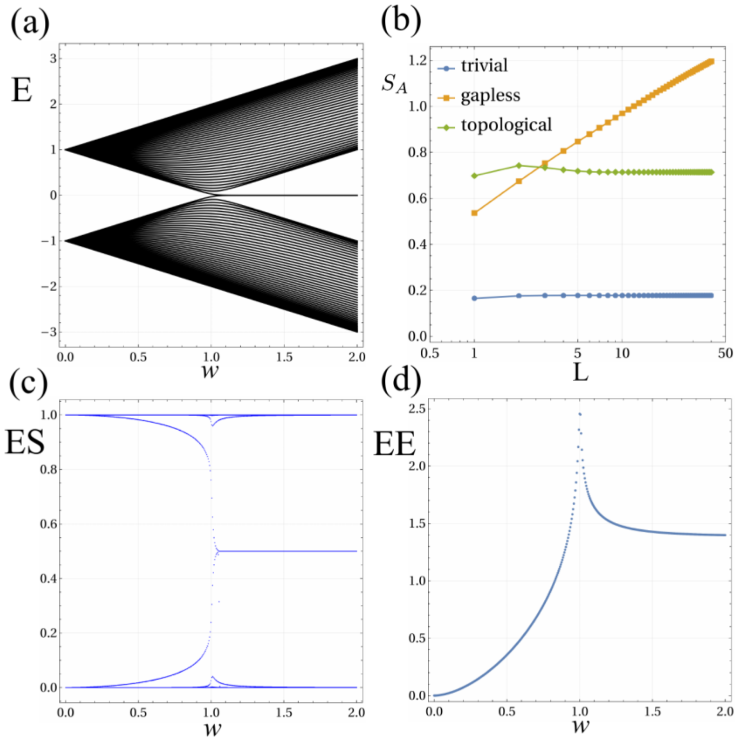

Supplementary Figure 11 | a, Energy spectrum of the SSH model with open boundary condition. **b**, The scaling behaviors of the entanglement entropy in different phases where the horizontal axis is scaled to be logarithm of L. **c**, The numerical simulation of the entanglement spectrum versus w. **d**, The numerical simulation of the entanglement entropy versus w.

Next, we move on to the scaling behaviors of the entanglement entropy in one-dimension gapless free-fermion systems. Considering a 1D free-fermion system with the energy dispersion shown in Supplementary Fig. 10, there are two lower-energy chiral gapless modes with left and right velocities. In the thermodynamical limit, the entanglement entropy of this 1D gapless system is represented as[9,14]

$$S_A = \frac{c}{6} \log L_A + \ldots, \quad (40)$$

where $c = c_L + c_R$ is the total central charge of the underlying conformal field theory, $c_L (c_R)$ is the chiral central charge of the left (right)-moving gapless mode, and L_R is the length of the subsystem A. The symbol … denotes all non-universal

contribution[9,14]. The scaling behavior of the entanglement entropy in higher-dimensional free-fermion gapless systems, related to the Widom conjecture, can be regarded as a generalization of Eq. (40). Furthermore, the scaling of entanglement entropy at the 1D conformal critical points is also described by Eq. (40)[15].

For numerically studying the entanglement entropy and entanglement spectrum in 1D case, we focus on the 1D Su-Schrieffer-Heeger (SSH) model written in the momentum space:

$$H_{SSH}(k) = \begin{pmatrix} 0 & we^{-ik} + v \\ we^{ik} + v & 0 \end{pmatrix} - \mu\sigma_0, \tag{41}$$

where the two continuous parameters $w, v \in \mathbb{R}$ and k denotes the 1D momentum. σ_0 denotes the two-dimensional identity matrix. By diagonalizing this Hamiltonian matrix, we arrive at the energy dispersion: $E(k) = \pm\sqrt{w^2 + v^2 + 2wv\,cos(k)}$. It is well known that, the SSH model has two distinct phases, namely topological phase and trivial phase. As shown in Supplementary Fig. 11a, the region $|v| < |w|$ is the topological phase with a finite bulk gap and with symmetry-protected gapless boundary modes, while the region $|v| > |w|$ is a trivial insulating phase. A topological phase transition occurs at the critical points $|v| = |w|$ where the bulk gap is closed.

Next, we study the entanglement entropy of the SSH model in two distinct phases as well as the critical points. As shown in Supplementary Fig. 11b, the entanglement entropy of topological and trivial phases, both of which are gapped, saturates to a constant at the scaling limit. Meanwhile, the SSH model at the critical point $w = v$ is a gapless free-fermion system in which the entanglement entropy has a logarithmic scaling behaviour $S_A \sim \frac{c}{6}\log L$, where c is the total central charge. Additionally, the set of points at the critical point $v = w$ in Supplementary Fig. 11b is fitted as $1.19 + 0.33\log L$, we find that the central charge of the critical point $w = v$ approximately equals to $c = 2$. We also study the entanglement entropy by tuning w but fixing L. We find that the entanglement entropy of the SSH model reaches a sharp peak located at the critical point, as shown in Supplementary Fig. 11d.

Meanwhile, the entanglement entropy in the topological phase has a lower bound of "2log2"[16], as shown in Supplementary Fig. 11d. In Supplementary Fig. 11c, we demonstrate the entanglement spectrum of the SSH model and find there exist $\frac{1}{2}$ modes in the entanglement spectrum, which shows an exact correspondence to the topological boundary states[12]. Furthermore, for the experimental simulation of the phononic system, the system usually satisfies open boundary condition. Then, we should consider the effect of the system's boundary for the entanglement of the SSH model. As shown in Fig. 12a, we adopt two kinds of partitions which locate in the middle and boundary of the system and are called Middle Partition and Boundary Partition, respectively. By using the tight-binding calculation, the entanglement spectrum of two partition are obtained in Fig.12b and c. We find that in the topological phase of the SSH model, the entanglement spectrum of Boundary Partition has only one $\frac{1}{2}$ mode in Fig. 12b, compared with the entanglement spectrum of Middle Partition in Fig. 12c. Then, we propose that the number of $\frac{1}{2}$ modes in the entanglement spectrum of Boundary Partition would decrease the lower bound of entanglement entropy to "log2" in the topological phase. Moreover, we show the entanglement entropy with two kinds of partitions in trivial, gapless and topological phases to study the effect of the location of the subsystem. As shown in Fig. 12 d-f, the behaviours of the entanglement entropy in three cases are not affected by the location of the subsystem, while the location of the subsystem only quantitatively changes the value of the entanglement entropy.

Besides, to consider the effect of the symmetry and the edge states for the lower bound of EE in topological phase, we introduce the next-nearest neighboring hoppings and add a constant potential on the boundary lattice sites of the SSH model to remove the edge states, while the modified SSH model breaks chiral symmetry and has inversion symmetry. Although the edge states is disappeared in Fig. 12g, we consider that the bulk states of the model can be still topological with non-trivial Zak phase. Meanwhile, we find that the EE of the modified SSH model in topological

phase still has the lower bound of "2log2" in Fig. 12h by adopting Middle Partition in Fig. 12a[16], where the lower bound remains unaffected by the presence or absence of edge states, indicating that it solely depends on the topology of the bulk states. For the ES in this case, the $\frac{1}{2}$ modes are still existed in Fig. 12i, which also shows the topology of the bulk states.

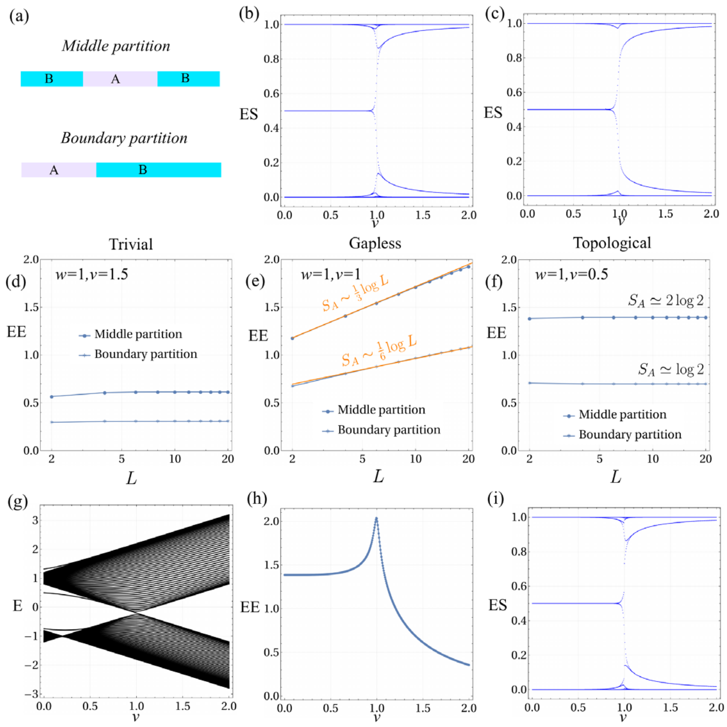

Supplementary Figure 12 | a, Two kinds of partitions for the SSH model. **b** and **c** is the entanglement spectrum of two partitions for the SSH model, respectively**. d**, The scaling behaviours for the two partition schemes for the trivially gapped phase in 1D. **e**, The scaling behaviours for the two partition schemes for the gapless phase in 1D. **f**, The scaling behaviours for the two partition schemes for the topologically gapped phase. **g**, Energy spectrum of the tight-binding SSH model breaking chiral symmetry with open boundary condition. **h**, The numerical simulation of the entanglement entropy for SSH model breaking chiral symmetry versus v. **i**, The numerical simulation of the entanglement spectrum for the modified SSH model breaking chiral symmetry versus v. For all the calculations of **g-i**, the filling of the valence band is considered. The entire system has 80 unit-cells, while the subsystem has 40

unit-cells for all cases. For the modified SSH model, the next-nearest-neighbor hopping is 0.2, and the onsite potential for the two unit-cells at the left and right edge boundaries are 1.

Supplementary Note 7: Probing band topology without relying on bulk-boundary correspondence and reliable indication of the topological transition even at small subsystem sizes

The acoustic crystal studied here lacks the chiral symmetry due to the non-negligible couplings among next-neighbor cavities, which, under specific geometric parameters, may render the supposed topological mid-gap end states to merge into the bulk continuum, leading to the breakdown of the bulk-boundary correspondence. Indeed, the bulk-boundary correspondence in our acoustic SSH model is broken, as signified by the eigen spectra of a finite lattice as a function of r_1 shown in Supplementary Fig. 13a. As we can see, in the topological phase ($r_1 < 4$mm), due to the absence of chiral symmetry, the end states labeled by the red curve only manifest at lower r_1, while merging into the bulk when r_1 increases. We can further alter the geometric parameters, for example, taking the geometric parameters of cylindrical cavities as $h = 50$mm and $d = 18$mm, to further break the chiral symmetry and thereby have no end states in the topological phase despite the variation of r_1 (see Supplementary Fig. 13b). In this case, the bulk-boundary correspondence is completely broken, while the corresponding entanglement entropy and entanglement spectrum still indicate the phase transition and exhibit the quantized value of $2log2$ and 0.5 (see Supplementary Figs. 13c and 13d), respectively, in the topological phase.

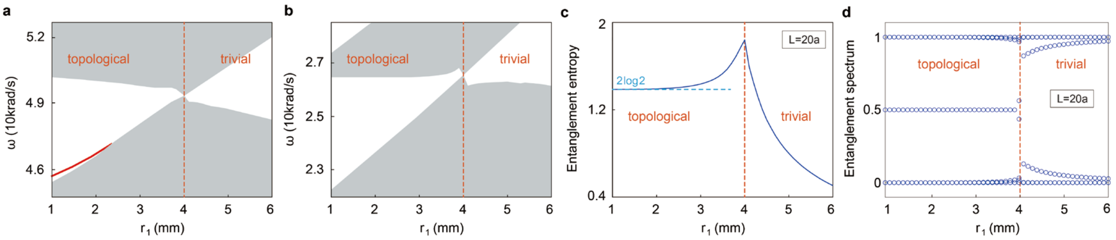

Supplementary Figure 13 | a, Eigen-spectrum of finite phononic crystals (with 40 unit-cells) as a function of the radius of the intra-unit-cell coupling tube r_1. In the topological phase (r_1<4mm), due to the absence of chiral symmetry, the edge states (labeled by the red curve) disappear for the topological phase with 2.4mm< r_1 <4mm, while they appear only for r_1<2.4mm. Here the other geometry parameters are the same as in the main text. **b**, By changing the parameters of the cylindrical cavities as h=50mm and d=18mm, the chiral symmetry is broken more severely, leading to the absence of the edge states in the topological phase in the whole range of 1mm<r_1<4mm. For the cases in **b**, the topological phase can still be faithfully identified via the entanglement entropy and entanglement spectrum. **c**, Entanglement entropy as a function of the radius r_1 clearly indicates the topological transition at r_1=4mm and gives the topological entanglement entropy of 2log2 in large gap cases (i.e., small r_1 cases). **d**, Most importantly, the entanglement spectrum changes sharply at the topological transition. In the topological phase (almost for the whole parameter range with r_1<4mm), a gap and a branch at 0.5 emerges in the entanglement spectrum which is a clear and faithful indication of the topological phase even in the absence of the chiral symmetry. These results demonstrate clearly the power of the entanglement entropy and entanglement spectrum in identification of topological phases.

We also do some simulations to explore the entanglement entropy and entanglement spectrum with lower L for the subsystem. As we can see from Supplementary Fig. 14, both entanglement entropy and entanglement spectrum can still faithfully indicate the topological phase. However, due to the finite size effect, the topological transition becomes less sharp in both entanglement entropy and entanglement spectrum for small subsystem sizes. Nevertheless, for $L \geq 6a$, the entanglement entropy shows a notable peak at the topological transition, while the entanglement spectrum exhibits an abrupt change at the same transition point at $r_1 = 4$ mm. These results indicate that both entanglement entropy and entanglement spectrum can give reliable indication of the topological transition even at small subsystem sizes.

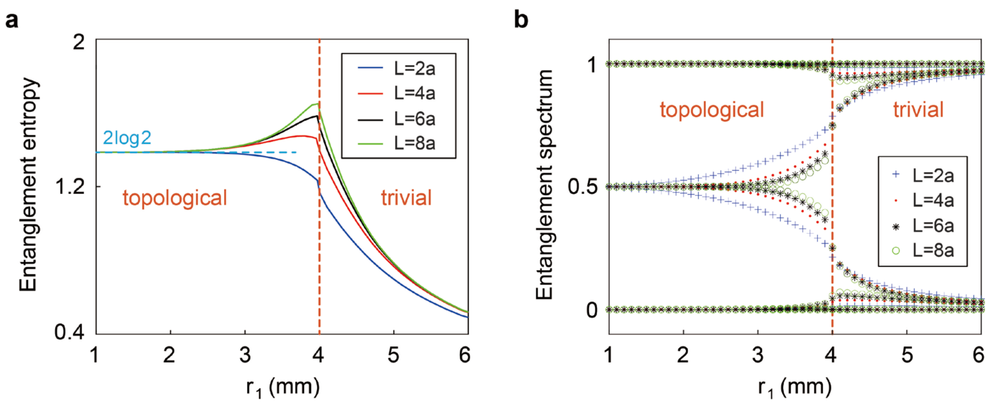

Supplementary Figure 14 | a, Topological transitions monitored by the entanglement entropy for various subsystem sizes. **b,** Topological transitions monitored by the entanglement spectrum for the same cases. All data from finite-element simulations of acoustic waves.

Supplementary Note 8: Details of the phononic edge dispersion measurement

To measure the phononic edge dispersion as shown in Fig. 4d in the main text, we insert the loudspeaker into a cavity around the middle of the zigzag boundary, the cavity situation, denoted by j_s, is marked by a green arrow in Supplementary Fig. 3b. The response function $\chi_{\alpha\beta}(i, j_s, \omega)$ is then detected within two zigzag lines near the edge boundary, which is found to be sufficient to extract the edge dispersion. After Fourier transformation of the response function, we obtain the dispersion of edge states along the zigzag direction.

Supplementary Note 9: Measurement of the Zak phase

The main text has shown that the band topology can be manifested by the entanglement entropy and entanglement spectrum. Conventionally, the band topology is also characterized by the Zak phase when the system respects inversion symmetry,

which gives quantized 0 or π corresponding to the trivial and topological bands. The Zak phase can be directly derived in our experiments, based on experimentally obtained wavefunctions. For the SSH lattice, the Zak phase for the lower phononic band is obtained through the discrete Wilson loop approach,

$$\nu = -Im\{ln[(\prod_{i=1}^{40}\langle u_{k_i}||u_{k_i+\delta k}\rangle)]\}, \quad (42)$$

where u_{k_i} is the experimentally obtained periodic part of the unit-cell Bloch wavefunction at Bloch wavevector k_i. $\delta k = 2\pi a/40$ due to the discretization of 40 non-equivalent points in the first Brillouin zone. $|u_{k_{40}+\delta k}\rangle = |u_{k_1}\rangle$. The Zak phases versus r_1 are presented in Supplementary Fig. 15a, showing a great consistence with the simulated values in both topological and trivial phases, as well as the topological transition at $r_1 = 4$mm.

For the honeycomb lattice in the gapless phase, the Bloch wavevector has two components k_1 and k_2. We can fix, for example, k_1, and then apply the discrete Wilson loop approach upon the Bloch wavefunctions along k_2. The Zak phases versus k_2 are shown in Supplementary Fig. 15b, which also agrees well with the simulated ones and implies the emergent topological edge states between two Dirac nodes. When the inversion symmetry is broken, as like the honeycomb lattice in the gapped phase, the Zak phase is no longer quantized and thus lose its topological characterization.

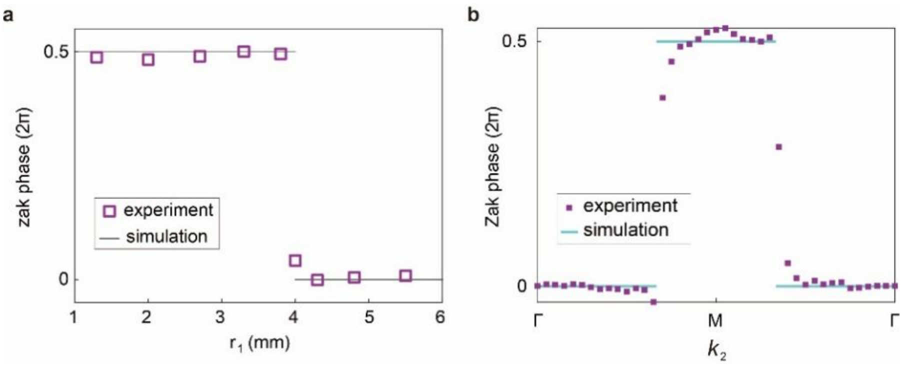

Supplementary Figure 15 | Experimentally obtained Zak phases of the SSH lattices (**a**) versus r_1, and the honeycomb lattice in gapless phase (**b**) versus k_2. They nearly coincide with the theorical values of quantized 0 or π, and show topological phase transitions at $r_1 = 4\,mm$ in (**a**) and at two Dirac nodes in (**b**).

Supplementary References

[1] Peschel, I. Calculation of reduced density matrices from correlation functions. *J. Phys. Math. Gen.* **36**, L205–L208 (2003).

[2] Klich, I. Lower entropy bounds and particle number fluctuations in a Fermi sea. *J. Phys. A: Math. Gen.* **39**, L85–L91 (2006).

[3] Cheong, S.-A. & Henley, C. L. Many-body density matrices for free fermions. *Phys. Rev. B* **69**, 075111 (2004).

[4] Huang, Z. & Arovas, D. P. Entanglement spectrum and Wannier center flow of the Hofstadter problem. *Phys. Rev. B* **86**, 245109 (2012).

[5] Lee, C. H., Ye, P. & Qi, X.-L. Position-momentum duality in the entanglement spectrum of free fermions. *J. Stat. Mech.* 2014, P10023 (2014).

[6] Gioev, D. & Klich, I. Entanglement Entropy of Fermions in Any Dimension and the Widom Conjecture. *Phys. Rev. Lett.* **96**, 100503 (2006).

[7] Widom, H. On a class of integral operators on a half-space with discontinuous symbol. *J. Funct. Anal.* **88**, 166–193 (1990).

[8] Leschke, H., Sobolev, A. V. & Spitzer, W. Scaling of R\'enyi Entanglement Entropies of the Free Fermi-Gas Ground State: A Rigorous Proof. *Phys. Rev. Lett.* **112**, 160403 (2014).

[9] Calabrese, P. & Cardy, J. Entanglement entropy and conformal field theory. *J. Phys. A: Math. Theor.* **42**, 504005 (2009).

[10] Li, W., Ding, L., Yu, R., Roscilde, T. & Haas, S. Scaling behavior of entanglement in two- and three-dimensional free-fermion systems. *Phys. Rev. B* **74**, 073103 (2006).

[11] Barthel, T. & Miao, Q. Scaling functions for eigenstate entanglement crossovers in harmonic lattices. *Phys. Rev. A* **104**, 022414 (2021).

[12] Fidkowski, L. Entanglement Spectrum of Topological Insulators and Superconductors. *Phys. Rev. Lett.* **104**, 130502 (2010).

[13] Zhou, Y. & Ye, P. Entanglement signature of hinge arcs, Fermi arcs, and crystalline symmetry protection in higher-order Weyl semimetals. *Phys. Rev. B* **107**, 085108 (2023).

[14] Calabrese, P. & Cardy, J. Entanglement entropy and quantum field theory. *J. Stat. Mech.* 2004, P06002 (2004).

[15] Vidal, G., Latorre, J. I., Rico, E. & Kitaev, A. Entanglement in Quantum Critical Phenomena. *Phys. Rev. Lett.* **90**, 227902 (2003).

[16] Sirker, J., Maiti, M., Konstantinidis, N. P. & Sedlmayr, N. Boundary fidelity and entanglement in the symmetry protected topological phase of the SSH model. *J. Stat. Mech.* 2014, P10032 (2014).
